# The Need for the Right Socio-Economic and Cultural Fit in the COVID-19 Response in Sub-Saharan Africa: Examining Demographic, Economic Political, Health, and Socio-Cultural Differentials in COVID-19 Morbidity and Mortality

**DOI:** 10.3390/ijerph17103445

**Published:** 2020-05-15

**Authors:** Andre M. N. Renzaho

**Affiliations:** 1 School of Social Sciences, Western Sydney University, Penrith 2751, Australia; andre.renzaho@westernsydney.edu.au; 2 Translational Health Research Institute, Western Sydney University, Penrith 2751, Australia; 3 Burnet Institute, Maternal, Child and Adolescent Health Program, Melbourne 3004, Australia

**Keywords:** coronavirus disease, COVID-19, sub-Saharan Africa, health systems, morbidity, mortality

## Abstract

The coronavirus disease (COVID-19) has spread quickly across the globe with devastating effects on the global economy as well as the regional and societies’ socio-economic fabrics and the way of life for vast populations. The nonhomogeneous continent faces local contextual complexities that require locally relevant and culturally appropriate COVID-19 interventions. This paper examines demographic, economic, political, health, and socio-cultural differentials in COVID-19 morbidity and mortality. The health systems need to be strengthened through extending the health workforce by mobilizing and engaging the diaspora, and implementing the International Health Regulations (2005) core capacities. In the absence of adequate social protection and welfare programs targeting the poor during the pandemic, sub-Saharan African countries need to put in place flexible but effective policies and legislation approaches that harness and formalise the informal trade and remove supply chain barriers. This could include strengthening cross-border trade facilities such as adequate pro-poor, gender-sensitive, and streamlined cross-border customs, tax regimes, and information flow. The emphasis should be on cross-border infrastructure that not only facilitates trade through efficient border administration but can also effectively manage cross-border health threats. There is an urgent need to strengthen social protection systems to make them responsive to crises, and embed them within human rights-based approaches to better support vulnerable populations and enact health and social security benefits. The COVI-19 response needs to adhere to the well-established ‘do no harm’ principle to prevent further damage or suffering as a result of the pandemic and examined through local lenses to inform peace-building initiatives that may yield long-term gains in the post-COVID-19 recovery efforts.

## 1. Introduction

The coronavirus disease (COVID-19) has spread quickly across the globe since being first reported in Wuhan, China on 31 December 2019. At the beginning of the pandemic, Asian countries, led by China, had the most number of confirmed cases of COVID-19 globally. By 22 February 2020, 13 of the 29 countries affected by the COVID-19 pandemic were in Asia, but China accounted for 98.6% of all confirmed cases globally [1] Since then, China has emerged from a 2 month containment phase and moved into the mitigation stage whilst the pandemic spread to wreak havoc on American and European societies [2]. It was not until the second week of March that Africa experienced a rapid spread of the COVID-19 pandemic. Martinez-Alvarez and colleagues [3] have observed that the pandemic started later in sub-Saharan Africa than other regions in the world due to limited international air traffic.

After the World Health Organisation declared COVID-19 outbreak a Public Health Emergency of International Concern on 30 January 2020, some countries put in place a number of COVID-19 prevention and containment measures. Some sub-Saharan African countries reacted quickly and decisively in implementing international guidelines to limit imported COVID-19 cases and to control, manage, and prevent spread of the pandemic. However, the continent faces local contextual complexities that require locally relevant and culturally appropriate COVID-19 interventions. COVID-19 prevention measures have been adopted globally without taking into account the differences in demographic, economic, political, and socio-cultural landscapes between nations and regions. The one-approach-fits-all response to the COVID-19 will have immeasurable negative impacts in sub-Saharan Africa. This paper examines demographic, economic political and socio-cultural differentials in COVID-19 morbidity and mortality.

## 2. Demographic Differentials in COVID-19 Morbidity and Mortality

High-income countries most hit by the COVID-19 pandemic have an older population, with a median age ranging from 47.1 years for Germany, 45.5 years for Italy, 42.7 years for Spain, 41.4 years for France to 38.1 years for the Unites States. In these countries, noncommunicable diseases (NCDs) such as cardiovascular diseases, cancers, stroke, and chronic respiratory diseases are the leading causes of death. NCD mortality increases with age, and account for three quarter of all deaths [4]. In the case of the COVID-19, older age is also a risk factor for mortality, with the patients’ mean age ranging from 47 to 48.9 years [5,6] In China, Verity and colleagues [7] found that the COVID-19 case fatality rate was 3·67%. When demography and under-ascertainment were adjusted for, it averaged 1.38% but found substantially higher rates in older age groups: 0.32% in those aged <60 years vs. 6.4% in those aged ≥60 years, up to 13·4% in those aged 80 years or older. However, in older people, comorbidities have been associated with deleterious health outcomes among COVID-19 patients. In the United States, evidence suggests that patients with underlying health conditions such as diabetes mellitus, chronic lung disease, and cardiovascular disease are at higher risk for severe COVID-19 and have poorer outcomes than persons without these conditions [8]. In China, using sample of 1099 patients to explore COVID-19 clinical progression, Guan and colleagues [6] reported that patients with severe disease tendered to be older than those with nonsevere disease by a median of 7 years, with those with the highest risk for severity and death being people over the age of 65 years and patients with underlying conditions such as chronic obstructive pulmonary disease, diabetes, hypertension, diabetes, cerebro-cardiovascular disease, and cancer. These findings are similar to those reported in another study by Guan et al. [5]. They found that the most prevalent comorbidity was hypertension (16.9%) and diabetes (8.2%), with 8.2% of the patients reporting two or more comorbidities. Patients with comorbidity had poorer clinical outcomes than those without any, especially those with chronic obstructive pulmonary disease, diabetes, hypertension and cancer.

In contrast, the leading cause of deaths in sub-Saharan Africa are communicable, maternal, neonatal, and nutritional (CMNN) diseases including HIV/AIDS, neonatal disorders, lower respiratory infections, diarrheal diseases and malaria (https://vizhub.healthdata.org/gbd-compare/). As indicated in the foregoing discussion, whilst COVID-19 case fatality rate increases with comorbidities and age, the continent has the youngest population on the globe, hosting 39 of the world’s 50 youngest countries (Table 1) [9,10]. The median age is below 20 years (averaging 19.7 years), which equates to half the median age for China and less than half the median age for high-income countries. Only 2.9% of its population is aged 65 years or older. Although data on the case fatality rate by age are not available at time of writing, the WHO COVID-19 Situation Report [11] indicates that as of 9 April 2020, a total of 6217 confirmed cases and 142 confirmed deaths were reported in 44 sub-Saharan African countries (excluding Sudan), giving a case fatality rate of 2.3%. COVID-19 coexists with CMNN, and severe forms will occur in younger patients. Given that sub-Saharan Africa bears a high mortality burden from HIV/AIDS, lower respiratory infections and malaria, these comorbidities are likely increase the mortality rate across all age groups.

## 3. Economic Differentials in COVID-19 Morbidity and Mortality

In high income countries like Australia, the economic response to the COVID-19 pandemic has been decisive: announcing economic packages supporting businesses to manage cash flow challenges and retain employees and the provision of financial support to individuals and households [13]. In order to keep Australians in jobs, the government introduced a wage subsidy program to support both businesses and employees, announcing a $130 billion “JobKeeper” payment package on 30 March 2020. This economic package is designed to support businesses affected by the COVID-19 pandemic to meet employees’ wages as part of wide-ranging job retention and economic stimulus strategies. Employers will receive $1500 per fortnight per eligible employee. Employees receiving a fortnightly salary of $1500 or more before tax will continue to receive their regular income according to their contractual agreements whilst those who receive a fortnightly salary of less than $1500 before tax will receive a minimum of $1500 per fortnight [13]. The Australian government has also introduced a number of economic packages to support individuals and households. Benefits include expanding social welfare payments and introducing a new, time-limited COVID-19 supplement at rate of $550 per fortnight over six months for both existing and new recipients of JobSeeker Payment, Youth Allowance Jobseeker, Parenting Payment, Farm Household Allowance and Special Benefit [13]. In addition, on offer are two separate $750 payments to social security, veteran and other income support recipients and concession card holders. Other economic stimuluses include relaxing superannuation laws to allow individuals affected by the COVID-19 pandemic to access a tax-exempt amount of up to $10,000 of their superannuation in 2019–2020 and a further $10,000 in 2020–2021 [13].

Other affected countries in Europe, Americas, Middle East, Asia and the pacific have put in place similar economic stimuluses [14]. For example, the European Central Bank launched a €750 billion Pandemic Emergency Purchase Programme on 18 March 2020 to allow members of the European Union to absorb the COVID-19 shock. The Pandemic Emergency Purchase Programme complements the Coronavirus Response Investment Initiative approved by the European Parliament and the Council which has been in force since 1 April 2020 including €37 billion directed at health care systems, small and medium enterprises, labour markets and other vulnerable parts of our economies, and a further €28 billion of structural funds for meeting associated expenditures. The focus is on fiscal spending for the containment and treatment of COVID-19, financial support for firms facing severe disruption and liquidity shortages or affected workers facing unemployment and income losses [15,16].

In contrast, due to limited fiscal capacity, sub-Saharan African countries cannot afford to put in place adequate economic stimulus packages to boost the economy, support businesses, and provide financial support to individuals and households. Fiscal measures put in place in countries such as Ghana, Kenya, Mauritius, Ivory Coast, Nigeria, Senegal, or South Africa have been very meagre and insufficient [14]. They are geared towards tax payment deferrals and reductions, loans, and moratorium on debt repayments, with little on offer for boosting and maintaining employment and supporting individuals and households through social protection and welfare programs [14]. The International Labour Organisation (ILO)’s 2017–2019 world social protection report paints a stark and shocking picture of the state of social protection policies in sub-Saharan Africa [17]. Social welfare and protection programs are very poor and coverage by statutory social security schemes is inadequate and only covers workers in the formal economy and their families, where nine out ten workers in both rural and urban areas hold only informal jobs [18]. The data also show that only 10% of the population are covered by at least one social protection benefit and the coverage of children receiving child cash benefit is very low, at just 13.1% [17]. The majority of women giving birth do not have access to maternity cash benefits, with less than 10% of women in employment covered for maternity benefits. The coverage of unemployment protection benefits and assistance is merely 5.6%. It is worth noting that only 22.7% of employees above statutory pensionable age receive a pension whilst the percentage of the labour force and working-age population who are active contributors to old age pensions is only 9% and 6.3%, respectively [17]. Worryingly, although 3.1% of the population are aged 65 or older, the public social protection expenditure on pensions and other benefits (excluding health) for persons above statutory pensionable age is a merely 1.6% of the GDP [13].

Over 70% of workers are in vulnerable employment (e.g., limited access to social protection schemes, no formal work arrangements, or lacked decent working condition.) and the informal economy (e.g., street vendors and informal hawkers, local convenience stores, taxi drivers, home-base workers, informal sector farmers) contribute 50–80% of gross domestic product, and 60–80% of employment and 90% of new jobs [18,19]. Studies on informal trade as well as cross-border trade in sub-Saharan Africa [20,21] suggest that informal trade accounts for 40% of the GDP and 55.7% of employment; whilst informal trading represents the most important source of employment for self-employed women, accounting for 60% of nonagricultural self-employment. Within the trading business, cross-border trading accounts the biggest share of employment among women. For example, in the western African region, women informal cross-border traders employ an average of 1.2 people in their home and support on average 3.2 children and 3.1 dependants; their contribution to the national GDP in terms of value added in trade, amounts to 64% in Benin; 46% in Mali and 41% in Chad [20]. Similarly, overall, informal cross-border trade accounts for 20% of the GDP in Nigeria and 75% of GDP in Benin. In the Southern African Development Community (SADC) region, informal cross-border trade amounts to $17.6 billion per year, which equate to 30–40% of the total intraSADC trade, and women make up about 70% of informal cross-border traders [20,21]. Therefore, these data illustrate how the stringent lockdown measures will have a devastating effect of the regional national economies and affect predominantly women due to disruptions to trade and supply chains.

Inadequate economic stimuluses in sub-Saharan Africa to support affected businesses and maintain employment mean that unemployment will increase and the COVID-19 pandemic is predicted to trigger the first recession over the past 25 years, with the economic growth declining from a modest 2.4% in 2019 to −5.1% in 2020 [22]. The region has a large discrepancy between the unemployment rate and total labour underutilisation. Labour underutilisation is more than three times as high as the unemployment rate due poor quality of available jobs and the lack of employment protection legislation [23]. It is estimated that the pandemic will cost the region $37–$79 billion in output losses for 2020 [22]. The COVID-19 pandemic will equally have a devastating effect on household welfare. Drops in commodity demand and associated decrease in terms of trade, combined with lower employment will translate into a pronounced welfare loss for households. The pandemic will also adversely affect nearly every sector of the economy such as agriculture and nontradable services, with agricultural production expected to contract between 2.6% and 7% in the case of trade blockages and a 13–25% decline in food imports due to higher transaction costs as well as reduced domestic demand [22].

## 4. Health Landscape in COVID-19 Morbidity and Mortality

In industrialised countries affected by the COVID-19 pandemic such as Australia, Canada, the United Kingdom, Spain, Italy, and the Nordic countries, there is an expanded role of the government in the provision of health care to ensure adequate universal health coverage [24,25,26]. The provision of health care services and access is not based on the purchase of health insurance, but rather based on residence rights through a single-payer health care in which the government pays for all health care costs [24,25,26]. In some other countries, there is a coexistence of private insurance and universal health care to ensure people have adequate access to care. The share of the GPD on health spending averages 8.8%, but is as high as 16.9% in the United States, 12.2% in Switzerland, 11.2% in Germany and France and 11% in Sweden and Japan [27]. These countries have health portfolio budget with budgetary flexibility to meet unforeseen challenges.

The same cannot be said for sub-Saharan Africa. Whilst the continent accounts for 25% of the global disease burden, it has only 3% of the world’s health workers, 1% of the world’s trained health workers deal with its disease burden, and less than 1% of the world’s health expenditure [28,29]. Political instability and flawed democracies lead to health care being neglected and not prioritized [29]. The region has low health coverage, poorly equipped health systems, and high financial hardship, suggesting that the COVID-19 pandemic and the post pandemic recovery may require serious reforms and a shift in health care prioritisation and financing. For example, with 42.5% of the population under the age of 15 years [30], a median dependency ratio of 82% (well above of the ‘dividend’ threshold of 60%) [31], and more than a fifth of its population (23.2%) undernourished [32], the coexistence of COVID-19 and CMNN will have a devastating effect on hunger and malnutrition in the long-term. Similarly, the continent has one of the highest levels of child malnutrition (stunting, wasting, and underweight) globally (Table 1) [12]. COVID-19 will heighten vulnerability to malnutrition in many ways: straining community livelihoods and services that affected communities provide, food shortage as a result of lockdowns and disruptions in global and national food systems, orphaning a large number of children as a result of COVID-19 related parental illness and death.

The health financing in the regions also remains inadequate. When measuring expenditure on health, four main sources of health spending can be tracked: prepaid private spending (health spending sources from nonpublic programs funded prior to obtaining health care including private health insurance and services provided for free by non-governmental agencies); out-of-pocket spending (payments made by individuals for health maintenance, restoration, or enhancement); development assistance for health (financial and in-kind resources transferred through multilateral, bilateral and nongovernment organisations and charitable foundations); and government health spending (spending for health care derived from domestic sources excluding out-of-pocket, prepaid private, and development assistance for health) [33]. In most sub-Saharan countries, governments’ share of health spending is estimated at just 33.7% (Table 1). Private personal health spending (out-of-pocket and pre-paid private spending) accounts for the greatest share of health spending, estimated at 44.2% of health spending; whilst the global development assistance for health accounts for the remaining 22.1%. Government spending projections indicate slower growth in the amount of pooled resources expected to be available per person, with the universal health coverage expected to come from increases in efficiency rather than government spending [33]. Given that out of pocket payments account for the biggest share of health spending, it makes COVID-19 health seeking behaviours a monumental task for communities, families and individuals.

Of particular concern is the fact that the region has the highest burden of HIV/AIDS and tuberculosis (TB) globally but spending on HIV/AIDS in the region is financed predominantly from external sources, with the development assistance for health accounting for 63.9% of all HIV/AIDS spending. Similarly, the Institute for Health Metrics and Evaluation [34] developed the healthcare access and quality index on a scale from 0 (worst) to 100 (best) based on death rates that could be avoided by timely and effective medical care. The median healthcare access and quality index in sub-Saharan Africa is just 32.2 (vs. a global index of 54.4 and an index of ≥89.8 for high income countries) (Table 1). The result suggests that both access to and quality of health care remain a significant challenge and the health system may not be adequately to meet the health needs of the population during the COVID-19 pandemic.

## 5. Political Differentials in COVID-19 Morbidity and Mortality

In most industrialised countries, an inadequate response to the COVID-19 pandemic may mean the end of the government at the next election. The pandemic has been unpredictable and represents a massive challenge for world leaders with potential severe consequences in the 2021 general, presidential or parliamentary elections in the United States, German, the Netherlands, Norway or Portugal. For most sub-Saharan countries, the COVID-19 pandemic provides leaders an opportunity to postpone elections, consolidate power through censorship, silencing critics, and bypassing the parliament to issue restrictive decrees and laws that may advance their political agendas. For example, Ethiopia postponed its elections due to the COVI-19 pandemic and many countries on the continent are contemplating postponing elections planned in 2020 and 2021 [35]. The South African government enacted regulations criminalizing disinformation on the COVID-19 outbreak, mirroring moves made by other countries such as China, Egypt, Azerbaijan, Russia, Iran, the Philippines, Honduras, Thailand, Cambodia, and Singapore [36,37]. In doing so, the South African government has effectively suppressed the right to freedom of opinion and expression. Emerging stories from sub-Saharan Africa suggest that there are cases of beating and harassing civilians such as targeting LGBT people, beating street fruit and vegetable sellers and motorcycle taxi riders, and killing people to enforce measures aimed at preventing the spread of COVID-19 [38]. Gift Trapence, a human rights defenders in Malawi’s Human Rights Defenders Coalition, summed up the situation in Malawi: ‘the government is happy with the coronavirus status and want to use it as a scapegoat to continue the president’s rule’ [38]. The use of oppressive law enforcement tactics and the oppression and exclusion of key institutions in a community that may hold different opinions or policy options such as religious groups, opposition leaders and political activists or civic organizations represent a threat to the fragile peace in the region.

The association between war and infectious diseases remains conflicting but the evidence points to wars and political instability exacerbating risk factors for the spread of infections. There is no doubt that sub-Saharan Africa remains the region in the world that has struggled to maintain political stability and has experienced sustained periodic spikes in political uncertainty and violence since the 1960s when many of its countries became independent from colonial rules. The ongoing violence in the eastern region of the Democratic Republic of the Congo, brutal war in South Sudan since its independence in 2011, war in Central African Republic, ethnic strife in Ethiopia, and Islamist insurgency in Burkina Faso, Northern of Nigeria, Somalia, and Northern Mali provide enough evidence of the challenges associated with addressing the COVID-19 pandemic in the region. Evidence suggests that infectious diseases worsen the conditions created by war, affecting both armed forces and civilians [39]. Wars and political instability lead to increased population displacements and associated overcrowding, lack of access to clean water and shelter, poor sanitation, poor nutritional status, and the collapse of public health infrastructure [39]. Similarly, war-related lack of health services hamper the effectiveness of control and prevention programs to reduce disease transmission [39]. Available data indicate that epidemics and pandemics decimate the fighting strength of armies, mainly due to the suspension and cancellation of military operations and wreak havoc among belligerent and nonbelligerent states [40]. Similarly, political conflicts, violence and wars lead to frequent displacements of troops and equipment as well as displaced persons, all of whom are more likely to carry COVID-19 pathogens. Controlling the spread on COVID-19 in sub-Saharan Africa may prove difficult in hot spots due to the combination of destroyed physical and health infrastructure and protracted cycles of violence and insecurity. Whilst governments may use the COVI-19 pandemic to maximise military and political gains by making upcoming elections a contested nightmare, insurgency groups may as well use the opportunity to create political havoc and make the response to the COVI-19 pandemic a logistical nightmare.

## 6. Socio-Cultural Differentials in COVID-19 Morbidity and Mortality

Strategies to halt the spread of the COVID-19 pandemic have incorporated three pillars: personal and respiratory hygiene, social distancing and lockdowns. Personal hygiene has remained an important protection strategy against COVID-19, and personal hygiene messages have reinforced the need to wash hands regularly and thoroughly with either an alcohol-based hand rub or soap and water. Implementing such a strategy in sub-Saharan Africa represents a challenge because of the lack of basic infrastructure such as piped water, sewage, or environmentally sound landfills. For example, the proportion of municipal solid waste collected regularly in only 32%, but this does not guarantee that solid waste is disposed properly, as most municipal solid waste disposal are open dumpsites, which contribute significantly to water pollution [41]. A staggering 42% of people are without a basic water supply whilst 72% are without basic sanitation [42]. Only 15% of the population have access to handwashing facilities with soap and water [43]. Around 40% of population live in urban cities, but two thirds (63%) of people in urban areas lack access to handwashing [44], less than 25% have access to piped water and only 35% use a flush toilet [45]. Additionally, 72% of them reside in deprived urban areas outside the largest city [45] whilst 55% of urban residents live in slums with living conditions characterized unfavourable environmental conditions such overcrowding and insecure tenure [46].

Similarly, the World Health Organisation, and government and nongovernment organisations have been providing guidelines for social distancing as a way to halt the spread of the COVID-19 pandemic. In collective cultures such those observed in sub-Saharan Africa, social support networks are key aspects of the patients’ care and recovery, characterized by health provider–relative–patient relationships which underscore the interplay between culture, context, and value systems in patients’ care expectations [47,48,49]. Social distancing and self-isolation are more likely to create social isolation, loneliness, and stigmatisation in this context. For example, anecdotal evidence emerging from the regions indicates that in some communities from low-income areas, people who have recovered from COVID-19 or have been discharged from government quarantine and hospitals face communal violence and are chased away from their homes by their neighbours due to limited understanding of the disease or just fear [50].

In turn, social isolation and loneliness are associated with deleterious health effects including increased likelihood of mortality by 29% and 26%, respectively [51], and increased risks of poor mental and cardiovascular health, musculoskeletal disorders, unhealthy lifestyles, and using psychotropic medications [52,53]. The construct of social isolation is incompatible with most sub-Saharan African cultures that emphasise and thrive on the collective. For example, when a patient is admitted to hospital, a close relative becomes a caregiver and stays in hospital with the patient to ensure appropriate care is given to the patient; the situation is aggravated by a severe deficit of professional health care givers in health institutions [48,49].

Besides, culture also matters and social relations are more likely to be characterised by unequal power in collectivist cultures than in individualist cultures [54,55,56]. Individualistic orientations predominate in most industrialised countries and the emphasis is on valuing personal space, privacy, independence, self-sufficiency, autonomy, and freedom [54,55]. The implementation and enforcements of compliance with social distancing measures must take into consideration personal freedom and rights. In these societies, people uphold their personal rights to emphasise the equal distribution of power where by the compliance to social distancing measures in place becomes voluntary and places great value on personal responsibility, control, and sovereignty [55]. In contrast, in sub-Saharan African communities, the collective and interdependence predominate [57]. People do not have personal space and are not self-sufficient. When one falls sick at home, their friends and relatives are expected to visit during the period of sickness. The most important relationships are vertical characterised by greater respect for authority and hierarchy, hierarchical decision-making, interdependence, obligations to the collective, adherence to and maintenance of traditional values and practices, and collective living arrangements [58]. In addition, there is a coexistence of common law and customary law [59]. Therefore, the unequal distribution of power means that existing laws favour some and the compliance to social distancing measures in place is coerced through state-sanctioned brutality and violence, and places great value on the state’s responsibility and power. Hence, social distancing interventions need to be specifically set and seriously take into account cultural diversity and contexts.

Many countries around the world have gone into lockdown to battle COVID-19 pandemic and restrictions for nonessential gatherings have been put in place. Briefly, the restrictions accepted in most industrialised countries mandate people to stay at home and to only leave their home if absolutely necessary such as to shop for food and essential supplies, attend medical appointments including compassionate visits, exercise either alone or with others in compliance with the public gathering requirements, and work or study if such activities cannot be carried out from home. Whilst most gyms and indoor sporting venues have been closed, lockdown restriction policies have been guided by social distancing measures, and have varied from country to country. For example, groups of people undertaking outdoor exercises must keep a distance of two meters between them, and each group must be made of no more than two people in Australia and the United Kingdom, five or less people in Norway, or no more than ten people in Finland and Denmark. In the United States, the federal government has recommended public gatherings of no more than ten people but only few states have taken up this recommendation including Colorado, Idaho, Iowa, Maine, Nebraska, Virginia, Wisconsin and the District of Columbia. Other states have implemented their own policies, with public gathering restrictions varying from up to 100 people in New Mexico and Utah to 50 or less people Louisiana, Maryland, New Hampshire, New Jersey, and no more than 25 people in Massachusetts and Oregon. Similar to the United States, the Canadian federal government has instituted a ban on all gatherings of 50 or more people, but each province has implemented its own policy. In countries such as Sweden, there has not been any lockdown, although the government has limited social gatherings to 50 people as no enforcement is needed, and the government emphasises voluntary action and social obedience as means to achieve this objective.

Aggressive restrictions on daily life in sub-Saharan African paint a pretty grim picture of the lockdown’s failures. Shopping for food and essential supplies or attending medical appointments including compassionate visits is a challenge as only 18% of the population have convenient access to public transport [41]. With the continent recording just 2% of vehicle ownership per capita [60], informal transport modes such as private minibuses and commuter taxis, motorcycles and scooters, and autorickshaws are widely used as alternative modes of transportation, but these have been banned. Walking and cycling remain among the main means available for the majority of city commuters to go shopping or to work, yet the movement of people has been prohibited. In countries such as Uganda, bans have been excessive and lockdown restrictions are complemented by a 7pm curfew. Measures have included banning public and private transportation, restricting markets to selling only foodstuffs, shutting down shopping malls and arcades, prohibiting of people movement, and a freeze on private car use [61]. South Africa also put in place some of the most stringent lockdown restrictions on the continent, banning jogging outside, the sale of alcohol or cigarettes, dog-walking, and citizens from leaving homes except for essential trips and prison [62]. In addition to these restrictions, outdoor exercise represents also a challenge as the majority of sub-Saharan African cities do not have a system of open public spaces that covers entire urban areas [41]. These aggressive measures could lead to more deleterious health effect, heightened poverty, high level of malnutrition, and increased domestic violence.

For example, data by the International Labour Organisation [18,19] indicate that prior to the COVID-19 pandemic, creating productive jobs was a key challenge for many sub-Saharan African countries and most employment is in the informal economy. It is this informal economy that has been hit hard by the lockdown. The prohibition of people movement and the use of personal modes of transport will mean loss of income and limited access to food and essential supplies. Similarly, lockdown restrictions include school closures, which in many may increase violence against children. For example, during the Ebola outbreak in Western Africa, school closures were associated with a significant increase in child labour, neglect, sexual abuse, and teenage pregnancies [63]. This is more likely to be the case during the COVID-19 pandemic. The increase in poverty, loss of control, social isolation, and uncertainty over the future brought about by the lockdown and subsequent school closures will likely translate into increased violence against children and gender-based violence. The number of women ostracised or kicked out of the house by their husbands and family-in-laws on suspicion of their COVID-19 status will increase [64,65].

## 7. Conclusions

Crippling healthcare expenditure, limited fiscal capacity, and stringent lockdown measures will make it difficult for sub-Saharan African countries to protect lives. Governments will be unable to inject much needed cash into the financial system to boost economies and increase the demand for goods and services. Reviving the informal economic sector to stimulate job creation and safeguard self-employment and entrepreneurship as well as employment and income for workers will remain a huge and long-lasting challenge. Although the world has adopted a one-size-fits-all approach, sub-Saharan Africa needs tailored COVID-19 interventions with the right socio-economic and cultural fit. The COVID-19 prevention and control strategies were formulated from an individualistic point view underpinned by individual freedom and right, with a long-term view of maintaining well-functioning health, social and economic systems. However, the stringent lock-down measures have forced individualistic cultures to be interdependent—the core value of collectivism. Collectivism has played an indispensable role in the prevention and control of the COVID-19 in Singapore, Japan, and China. Instead of harnessing the inherent collectivist values, many sub-Saharan African countries implemented draconian lockdown measures with no long-term objectives for the recovery of the severely affected social and economic systems. There was an opportunity for sub-Saharan African leaders to harness the cohesiveness among individuals and social hierarchies of collectivism to promote commitments to social norms and to effectively mobilize the collectivist spirit through the use of inherent social hierarchies as credible voices to enforce public health messages.

Although draconian measures in the fight of the COVID-19 pandemic may have delivered short term gains, it is the long-terms consequences that will severely affect the continent’s ability to recover. For example, given that the informal sector, especially trade and in particular cross-border trading, is the main source of income and a significant contributor to national economies, sub-Saharan African countries cannot afford to maintain stringent lockdown measures over a long period of time. Urgent alternative options are needed to swiftly enable trade facilitation and reduce trade and supply chain barriers. Similarly, the COVID-19 has hampered production globally, disrupted supply chains, and upset markets; affecting severely major donors to sub-Saharan Africa. Reduced donors’ capacity, weakened financial and health systems, fragile political stability, and reduced state capacity will create and perpetuate poverty and even reverse decades of development gains. To prevent such a disaster, drastic measures need to be put in place. First, there is an urgent need to strengthen the health systems and capacities to respond to emerging infectious disease outbreaks. This can be achieved by extending the health workforce through the use of social hierarchies, mobilizing and engaging the diaspora, and implementing the International Health Regulations (2005) core capacities. Second, in the absence of adequate social protection and welfare programs targeting the poor during the pandemic, sub-Saharan African countries need to put in place flexible but effective policies and legislation approaches that harness and formalise the informal trade and remove supply chain barriers. This could include strengthening cross-border trade facilities such as adequate propoor, gender-sensitive, and streamlined cross-border customs, tax regimes, and information flow. The emphasis should be on cross-border infrastructure that not only facilitates trade through efficient border administration but can also effectively manage cross-border health threats. Improved information flow could facilitate an effective monitoring of COVID-19 modes of transmission (international arrivals vs. community transmissions) and the test-track-trace strategy which encompasses identifying cases, undertaking a comprehensive contact tracing, and quarantining of contacts. Third, using legislative frameworks to strengthen and making social protection systems responsive to the crisis, and embedding them within human rights-based approaches could better support vulnerable populations and enact health and social security benefits. Fourth, the COVID-19 response needs to adhere to the well-established “do no harm” principle to prevent further damage or aggravate suffering as a result of the pandemic. Finally, the COVID-19 response needs to be examined through conflict lenses to address the political instability in the region. Political solutions to conflicts as well as peace-building approaches may yield sustainable and long-term gains in the post-COVID-19 recovery efforts.

## Figures and Tables

**Table 1:** Key health indicators in selected sub-Saharan African countries.

Country	Population [9,10]		PEM [12]			U5M [10]	HAQ [10]	Health Spending in USD [10]					Share of Health Spending		
	Million	Median Age	Stunting	Wasting	Under-weight			PPS	OPS	GHS	DAH	Total	Government Share	Private Spending	*DAH*
Angola	28.2	15.9	37.6	4.9	19.0	71.0	33.4	20	38	58	4	120	48.3%	48.3%	3.3%
Benin	11.6	18.2	34.0	5.0	18.0	89.8	30.8	2	14	7	9	32	21.9%	50.0%	28.1%
Burkina Faso	21.1	17.3	34.6	15.5	25.7	115.6	30.1	2	13	13	8	36	36.1%	41.7%	22.2%
Cameroon	27.8	18.5	32.5	5.6	14.6	78.7	31.9	1	43	9	5	58	15.5%	75.9%	8.6%
CAR	4.6	19.7	40.8	6.6	23.5	131.1	18.6	0	8	3	11	22	13.6%	36.4%	50.0%
Chad	15.2	17.8	39.9	13.0	28.8	122.8	25.4	2	21	8	5	36	22.2%	63.9%	13.9%
Congo	4.9	19.7	21.2	8.2	12.3	59.9	34.1	3	35	37	3	78	47.4%	48.7%	3.8%
Cote d’Ivoire	25.0	18.9	29.8	7.5	14.9	94.7	27.3	14	33	18	11	76	23.7%	61.8%	14.5%
Djibouti	1.1	23.9	33.5	21.5	29.8	48.8	35.0	1	16	35	15	67	52.2%	25.4%	22.4%
DRC	80.9	18.8	42.7	7.9	22.6	87.3	29.6	2	8	3	7	20	15.0%	50.0%	35.0%
Equatorial Guinea	1.3	19.8	26.2	3.1	5.6	59.2	49.3	13	222	67	9	311	21.5%	75.6%	2.9%
Eritrea	5.9	19.7	52.5	14.6	38.8	51.3	27.6	1	19	6	4	30	20.0%	66.7%	13.3%
Eswatini	1.1	21.7	28.9	2.5	5.4	49.9	40.5	24	32	199	74	329	60.5%	17.0%	22.5%
Ethiopia	102.9	17.9	40.4	8.7	25.2	60.6	28.1	5	11	7	8	31	22.6%	51.6%	25.8%
Gabon	1.7	18.6	16.5	3.3	5.9	40.5	40.4	35	69	175	3	282	62.1%	36.9%	1.1%
Gambia	2.1	19.9	24.5	11.5	16.2	51.2	35.7	2	5	5	16	28	17.9%	25.0%	57.1%
Ghana	30.2	21.1	18.8	4.7	11.0	62.8	39.3	5.3	31	29	9	74.3	39.0%	48.9%	12.1%
Guinea	11.8	18.9	31.2	9.6	18.0	103.1	26.4	4	24	5	11	44	11.4%	63.6%	25.0%
Guinea-Bissau	1.9	20.1	27.6	6.0	17.0	80.6	23.4	0	17	17	16	50	34.0%	34.0%	32.0%
Kenya	48.3	19.7	26.0	4.0	11.0	48.2	39.5	12	22	28	19	81	34.6%	42.0%	23.5%
Liberia	4.7	17.8	31.6	5.9	15.0	78.9	32.2	5	34	8	34	81	9.9%	48.1%	42.0%
Madagascar	26.1	19.7	41.6	6.4	36.8	78.1	29.6	2	6	11	4	23	47.8%	34.8%	17.4%
Mali	20.3	15.8	38.3	12.7	25.0	127.4	34.9	0	12	8	12	32	25.0%	37.5%	37.5%
Mauritania	3.9	20.5	22.8	11.5	24.9	49.3	40.6	2	28	20	5	55	36.4%	54.5%	9.1%
Mozambique	30.0	17.2	42.6	5.9	14.9	79.1	30.0	1	2	6	24	33	18.2%	9.1%	72.7%
Namibia	2.4	21.2	23.8	6.2	13.4	43.6	44.6	137	41	300	34	512	58.6%	34.8%	6.6%
Niger	21.4	15.4	43.9	18.0	36.4	112.0	28.4	1	15	7	4	27	25.9%	59.3%	14.8%
Nigeria	206.1	18.4	36.8	8.7	28.7	109.5	41.9	1	54	10	6	71	14.1%	77.5%	8.5%
Rwanda	12.6	19.0	37.9	2.2	9.3	50.7	36.0	5	4	16	19	44	36.4%	20.5%	43.2%
Senegal	14.7	18.8	26.5	10.1	17.7	53.1	31.1	5	34	21	9	69	30.4%	56.5%	13.0%
Seychelles	0.1	35.4	7.9	4.3	3.6	12.5	65.6	0	11	522	1	534	97.8%	2.1%	0.2%
Somalia	16.9	18.1	25.3	14.3	23.0	110.4	19.0	0	4	3	7	14	21.4%	28.6%	50.0%
South Africa	55.0	27.1	17.4	2.5	5.9	35.9	49.7	186	40	274	12	512	53.5%	44.1%	2.3%
Sudan	40.3	19.9	38.2	16.3	33.0	47.7	45.8	4	78	26	4	112	23.2%	73.2%	3.6%
Tanzania	54.0	17.7	34.4	4.5	13.7	65.2	33.9	1	9	14	17	41	34.1%	24.4%	41.5%
Togo	7.5	19.8	27.5	6.5	16.0	73.0	32.0	4	23	9	6	42	21.4%	64.3%	14.3%
Uganda	39.1	15.8	33.4	4.7	13.8	66.6	31.4	1	17	7	19	44	15.9%	40.9%	43.2%
Zambia	17.4	16.8	40.1	6.0	1.5	70.5	29.0	4	8	24	28	64	37.5%	18.8%	43.8%
Zimbabwe	14.7	20.0	32.0	3.0	9.7	60.9	31.2	10	28	48	20	106	45.3%	35.8%	18.9%

PEM—Protein energy malnutrition; U5M—under five mortality; HAQ—personal healthcare access and quality; PPS—prepaid private spending; OPS—out-of-pocket spending; GHS—government health spending; DAH—development assistance for health; CAR—Central African Republic; DRC—Democratic Republic of Congo.
